# Integration of dual-energy CT parameters and radiomics features for non-invasive prediction of *α*-SMA and CD8 + T cell in non-small cell lung cancer

**DOI:** 10.3389/fmed.2026.1792692

**Published:** 2026-03-11

**Authors:** Nan Jiang, Yan Zhang, Gang-Feng Li, Xiao-Yan Qu, Wen-Xiu Wang, Rong Hou, Hong-Juan Ma, Yang Yang, Ying Yu, Guang-Bin Cui

**Affiliations:** 1 Department of Radiology, Tangdu Hospital, Fourth Military Medical University, Xi’an, Shaanxi, China; 2 Functional and Molecular Imaging Key Lab of Shaanxi Province, Xi’an, Shaanxi, China; 3 Department of Pathology, Tangdu Hospital, Fourth Military Medical University, Xi’an, Shaanxi, China

**Keywords:** CD8 + T lymphocyte, dual energy CT, non-small cell lung cancer, radiomics, tumor microenvironment, *α*-SMA

## Abstract

**Background:**

The non-invasive characterization of the tumor microenvironment (TME) is essential for stratifying non-small cell lung cancer (NSCLC) patients who may benefit from immunotherapy. This study investigates a novel approach by integrating dual-energy CT (DECT) parameters with radiomics to quantitatively assess stromal fibrosis (via *α*-SMA area) and CD8 + T-cell infiltration.

**Methods:**

In this prospective study, 70 treatment-naive NSCLC patients were enrolled. Preoperative DECT scans were used to extract both DECT parameters and radiomics features. Corresponding surgical specimens were analyzed to determine the area percentage of *α*-SMA-positive stroma and the density of CD8 + T cells, with patients classified into high and low groups for each biomarker. After feature selection, models were constructed based on DECT parameters alone, radiomics features alone, and a combined feature set. Models were evaluated via 5-fold cross-validation.

**Results:**

For predicting high *α*-SMA expression, the integrated model combining DECT parameters and radiomics features demonstrated superior performance (AUC: 0.766) compared to models using either modality alone (DECT AUC: 0.670; radiomics AUC: 0.703). In contrast, for predicting CD8 + T-cell density, the DECT-only model (AUC: 0.715) performed comparably to the radiomics model (AUC: 0.695), with no significant gain from integration. Key discriminating features, such as normalized iodine concentration for *α*-SMA and spectral slope of K40-70 for CD8+, showed significant intergroup differences and plausible biological correlations.

**Conclusion:**

The integration of DECT and radiomics presents a feasible, non-invasive strategy to assess specific TME components in NSCLC, underscoring the complementary value of different imaging data types towards developing biomarkers for personalized oncology.

## 1 Introduction

Non-small cell lung cancer (NSCLC) remains a leading cause of cancer-related mortality world-wide, with limited improvements in survival despite advancements in therapies such as immune checkpoint inhibitors (ICIs) (1). Only 20 ~ 50% of patients can get sustainable benefit from immune therapies, while more than 60% of patients respond eventually develop acquired resistance or may develop severe immune-related complications (2). In sight of these challenges, it is significant to identify patients who are favorable to immunotherapy before treatment and to evaluate persistently during or after the treatment (3).

The efficacy of immunotherapy is critically dependent on the dynamic interplay between immune-activating and immunosuppressive components within the tumor microenvironment (TME) (4). Among these, tumor-infiltrating lymphocytes (TILs), particularly CD8 + T cells, serve as a key prognostic indicator and predictor of treatment response (5–7). Conversely, cancer-associated fibroblasts (CAFs) marked by *α*-smooth muscle actin (α-SMA), constitute a major immunosuppressive element. These cells promote immunosuppression by fueling tumor progression and forming a barrier against immune infiltration, with α-SMA expression levels correlating with their activity and tumor invasiveness (8, 9). Therefore, a precise assessment of these two components is essential. However, current methods relying on invasive biopsies are constrained by spatial heterogeneity and are unsuitable for serial monitoring. This underscores an urgent need for the development of reliable, non-invasive preoperative evaluation tools.

The clinically accessible, multi-parametric data from medical imaging, particularly computed tomography (CT), offer a powerful, non-invasive means for TME assessment and are already integral to lung cancer management (10). Building on this, dual-energy CT (DECT) as an advanced evolution of conventional CT, acquires spectral data at different energy levels. This allows for the precise quantification of tissue composition and heterogeneity, offering functional insights beyond anatomical imaging. These derived parameters, such as virtual monoenergetic images (VMI) and iodine concentration (IC) maps, have demonstrated utility in revealing the histological and biological characteristics of NSCLC or other diseases (11). However, identifying the complex elements of TME solely through these parameters remains challenging. To address this gap, radiomics has been introduced as a complementary tool to decode the TME by extracting high-dimensional, sub-visual features that may correlate with specific cellular components (12). While studies have begun to demonstrate the utility of DECT-based radiomics in lung cancer diagnosis (13), its application for predicting specific TME components in NSCLC remains largely unexplored. Notably, pioneering work in other cancers provides proof of principle, for instance, a DECT radiomics model successfully predicted CD8 + T-cell density and CAF activity in clear cell renal cell carcinoma (ccRCC) (14). Collectively, this supports the potential of integrating DECT’s functional spectral information with radiomics as a promising, non-invasive strategy for characterizing the TME in lung cancer.

Therefore, we hypothesize that integrating DECT parameters with radiomic features may enable the non-invasive characterization of the ECM and immune infiltration in NSCLC. This holistic approach addresses key clinical gaps by providing a reproducible and quantitative assessment of the TME. It has the potential to inform both immunotherapy stratification and novel strategies targeting the ECM, ultimately advancing toward more personalized treatment for NSCLC patients.

## 2 Materials and methods

### 2.1 Patients

**Figure 1:**
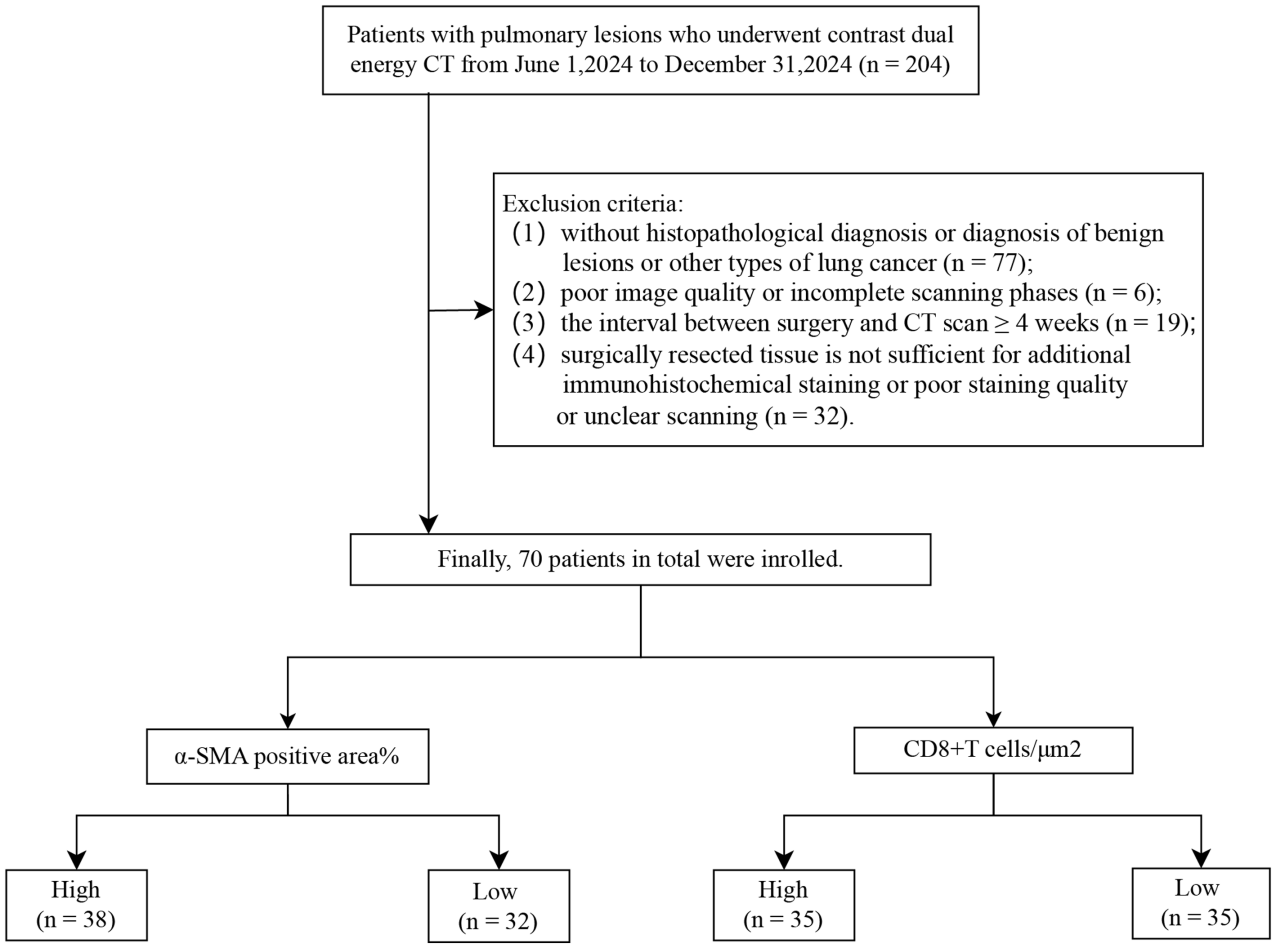
Enrollment flowchart of the study cohort.

The Institutional Ethics Committee of the local hospital approved this prospective study (K202504-75). The patients were enrolled from June 1st to December 31st 2024 with the inclusion criteria: (1) no prior treatment (including surgery, radiation, chemotherapy or immunotherapy) before DECT examination; (2) no concurrent active infections and any other malignant tumors; (3) availability of a complete routine blood test, including tumor markers, blood volume, platelet-to-lymphocyte ratio (PLR), and neutrophil-to-lymphocyte ratio (NLR), within 30 days prior to DECT examination. Initially, a total of 204 patients who underwent contrast-enhanced DECT examination were included. Patients were further screened according to the exclusion criteria detailed in the flowchart (Figure 1). Finally, 70 patients with pathologically confirmed NSCLC were enrolled in the final analysis.

### 2.2 DECT parameters acquisition

All examinations were performed on a third-generation dual-source CT scanner (Somatom Force, Siemens Healthcare). Detailed scanning protocols and acquisition parameters are provided in Supplementary S1.1. All images were transferred to a post-processing workstation (syngo.via; Siemens Healthineers) where two experienced radiologists (H* and Q**, respectively with 5 years and 10 years of chest radiology experience) measured DECT parameters independently on the largest slice at venous phase (VP) and delayed phase (DP) separately, avoiding necrosis, vascularity, and calcification without knowing the pathologic results. The average of the measurements from the two radiologists were used as the final result and the intra-class correlation coefficients (ICC) of all parameters were calculated.

Quantitative DECT parameters included monochromatic CT numbers at energy levels of 40, 70, 100 and 130 keV, effective atomic number (Zeff), electron cloud density (Rho), and IC. To reduce the impact of individual circulatory variation, the normalized iodine concentration (NIC) was calculated by dividing the lesion IC by the aortic IC (NIC = IC_lesion_/IC_aorta_). Aorta IC was measured by positioning a region of interest (ROI) within the aorta on the same slice, avoiding calcified plaques. If the aorta was not present on the same slice, the measurement was taken from the thoracic aorta. The slopes of the energy attenuation curves were calculated between different energy levels, including K40–70, K70–100 and K100–130. Finally, the ECV was derived using the formula: ECV (%) = (1 - hematocrit) × (IC_lesion_/IC_aorta_) × 100%, where IC_lesion_ and IC_aorta_ were the IC value of lesion and aortic at the DP, respectively.

### 2.3 Radiomics features extraction

Two experienced radiologists drew tumor borders manually in the largest cross-sectional area on 70 keV VMI at VP using 3D slicer software (^1^version 5.6.2) (15). Since previous studies have confirmed that the 70 keV VMI show the highest feature consistency with 120 kVp images (ICC = 0.98 ± 0.03) with better image quality (16). And during the VP, the contrast agent is fully distributed in the blood vessels and tissue stroma, allowing the tumor’s heterogeneity, boundaries, and internal structures to be displayed most clearly. Radiomics features were extracted by using the Pyradiomics package in python (version 3.7) (17). A total of 93 standardized quantitative features validated by the Image Biomarker Standardization Initiative (IBSI) were included (18). A random subset of 20 patients was selected by Q** using a computer-generated random number method after initial segmentation by H*. The ICC of all radiomic features was also calculated.

### 2.4 Pathological analysis

Specific antibodies, including CD8 (MXB-Bio mouse# MAB-1031) and *α*-SMA (MXB-Bio mouse# MAB-0890), were utilized for immunohistochemical staining. All the slicers were scanned using MoticEasyScan (Version 1.0). For each sample, the entire tumor section was first scanned at low magnification (40×) to identify regions with the highest apparent density of staining through MoticDSAssistant (Version 1.0). From within these hotspot regions, five non-overlapping ×200 magnification fields were then randomly captured for quantitative analysis. The number of CD8 + T lymphocytes as well as the proportion of positive area of *α*-SMA, were quantified using ImageJ (Version 1.54) (19) in 5 micrographs for each case and the results were evaluated by a pathologist with 10 years of experience, details described in Supplementary S1.2. The average of these values was calculated as the final quantity for each case. Subsequently, patients were dichotomized into high and low groups using the mean *α*-SMA area proportion (normally distributed) and the median CD8 + T-cell density (skewed distribution) as cutoffs (Supplementary S1.3, Supplementary Figure S3).

### 2.5 Feature selection

For DECT quantitative parameters as well as radiomics features, the feature selection was separately conducted through same steps. Firstly, all features were normalized, and only features demonstrating excellent inter-observer agreement (ICC > 0.75) were retained. To reduce redundancy, highly correlated features (Pearson correlation coefficient > 0.85) were filtered, retaining the feature with stronger relevance. Finally, least absolute shrinkage and selection operator (LASSO) regression was used for further dimensionality reduction.

### 2.6 Model construction and comparison

We constructed 6 models for differentiating each pathological subgroup, where DECT and radiomics model were constructed by logistic regression (LR) and combined models were constructed by LR, support vector machine (SVM), K nearest neighbors (kNN) and naive bayes (NB) using selected DECT and radiomics parameters. Following construction with logistic regression algorithm (LRA), all models underwent 10 times 5-fold cross validation (CV). The discriminatory performance was evaluated based on the receiver operating characteristics (ROC) curve reporting area under the curve (AUC), accuracy (ACC), specificity (SPE), sensitivity (SEN), positive predictive value (PPV), and negative predictive value (NPV). The prediction effect was evaluated using calibration curve with the overall performance was measured with Brier Score (BS). Decision curve analysis (DCA) was performed to evaluate the clinical utility of the prediction models as diagnostic tools. In this context, the ‘Treat-All’ strategy represents the net benefit of performing biopsies on all patients, while the ‘Treat-None’ strategy represents performing no biopsies. To evaluate how much each feature affects the model’s predictions, the SHapley Additive exPlanations (SHAP) was employed (20).

### 2.7 Statistical analysis

Statistical analyses were conducted by R software (Version 4.4.0). Continuous variables were summarized as mean ± standard deviation when normally distributed or as median (interquartile range) otherwise, with group comparisons conducted using t-tests or Wilcoxon rank-sum tests. Categorical variables were expressed as frequencies and percentages and compared using chi-square or Fisher’s exact tests. Statistical significance was defined as a two-sided *p* value of less than 0.05. Additionally, the Spearman correlations of NLR and PLR with pathological biomarkers were evaluated, and their predictive performance was assessed using the area under the curve (AUC) derived from univariate logistic regression.

## 3 Results

### 3.1 Patient demographics

**Figure 2:**
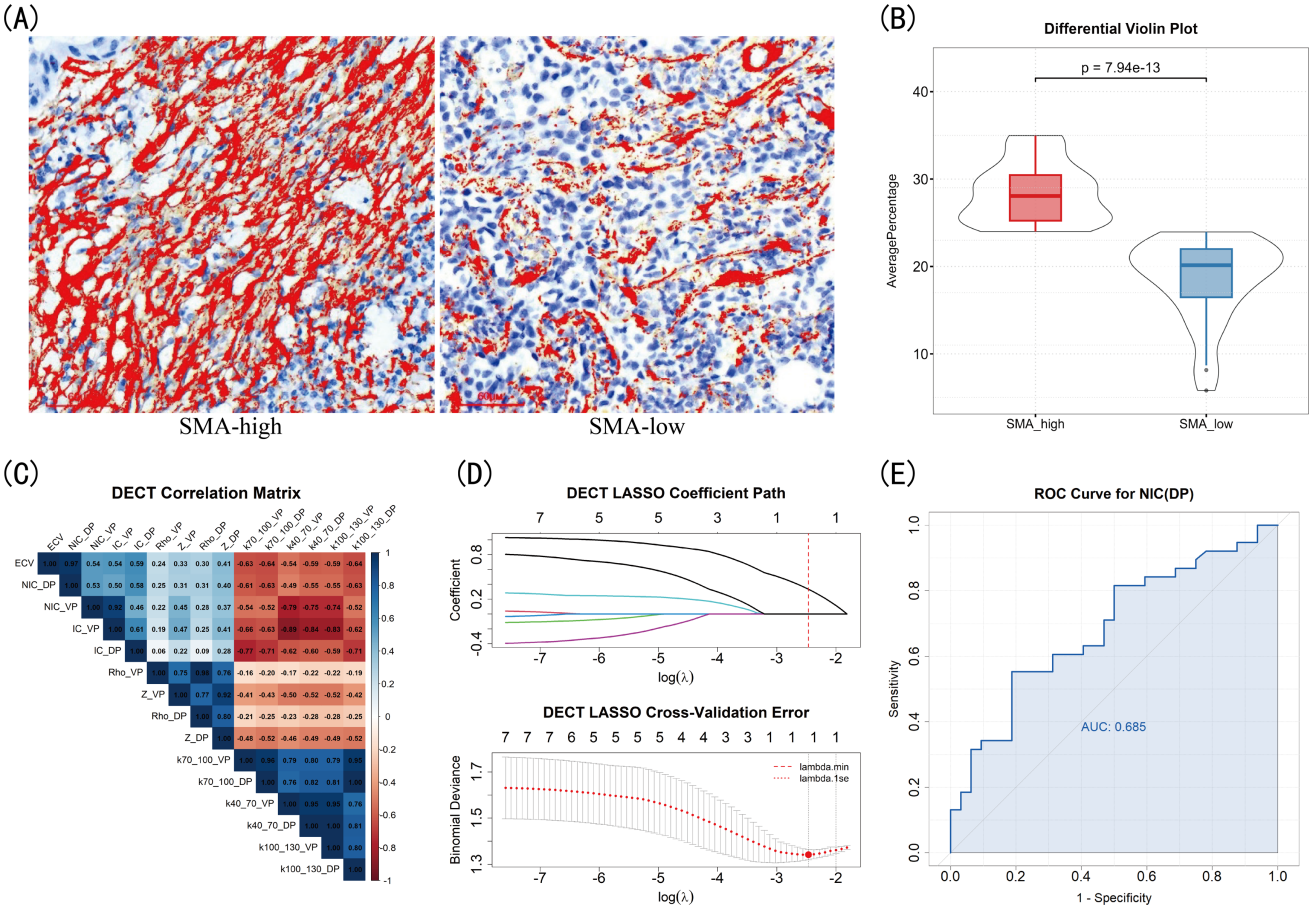
DECT parameters for differentiating SMA expression in NSCLC. **(A)** Immunohistochemical expression quantification. **(B)** The violin comparison plot. **(C)** Correlation heatmap. **(D)** LASSO selection plots. **(E)** ROC of selected parameters.

**Figure 3:**
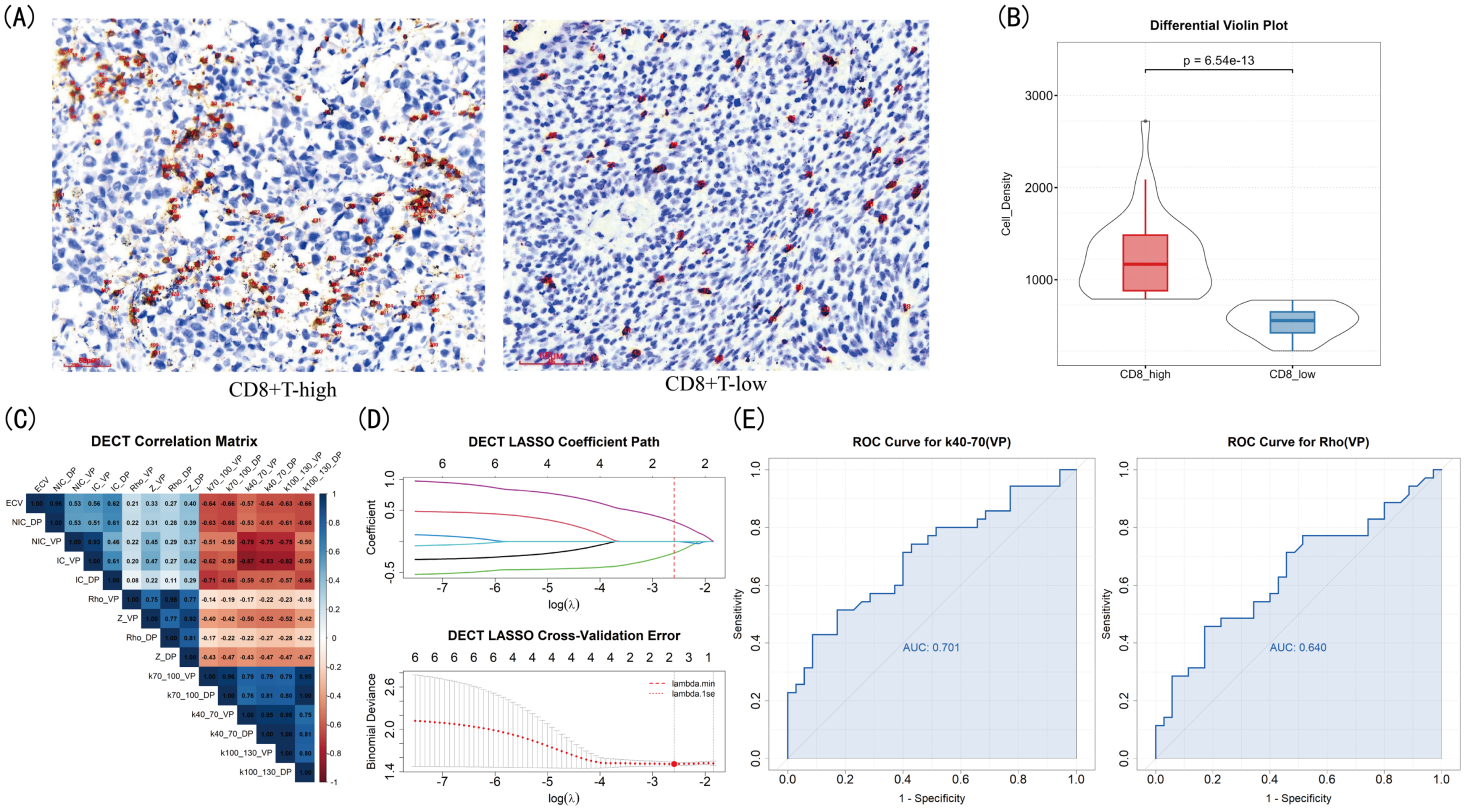
DECT parameters for differentiating CD8 expression in NSCLC. **(A)** Immunohistochemical expression quantification. **(B)** The violin plot. **(C)** Correlation heatmap. **(D)** LASSO selection plots. **(E)** ROC of selected parameters.

A total of 70 eligible NSCLC patients (37 male, 33 female) with a mean age of 63.5 years were finally enrolled. The baseline characteristics of the entire cohort are summarized in Table 1. IHC analysis demonstrated the expression levels of *α*-SMA (Figure 2A) and CD8 (Figure 3A). Using a mean α-SMA positive staining area threshold of 24%, patients were stratified into SMA-high (*n* = 38, 54%) and SMA-low (*n* = 32, 46%) groups. There was a significant difference in SMA expression between the two groups (*p* < 0.001, Figure 2B), and the SMA-high group was significantly associated with higher CEA levels and a greater frequency of positive family history. Using a threshold of median value 785 CD8 + T cells/μm2, (*n* = 35, 50%) patients were classified into CD8-high group and (*n* = 35, 50%) into CD8-low group. There was a significant difference in CD8+T cell density between the two groups (*p* < 0.001, Figure 3B), and there were no significant differences in clinical, pathological features between CD8 groups. The analysis of systemic inflammatory indices (NLR, PLR) showed no significant correlation with the target pathological biomarkers (Supplementary Table S1).

**Table 1:** Demographic and clinical characteristics of patients.

Variables	Total (*n* = 70)	SMA-low (*n* = 32)	SMA-high (*n* = 38)	Statistic	*P*	CD8-low (*n* = 35)	CD8-high (*n* = 35)	Statistic	*P*
Age (years), M (Q₁, Q₃)	63.50 (58.00, 69.00)	63.50 (57.00, 72.00)	63.50 (59.00, 68.75)	*Z* = −0.29	0.772	65.00 (61.00, 71.00)	63.00 (56.00, 68.50)	*Z* = −1.42	0.155
BMI (kg/m^2^), Mean ± SD	23.66 ± 3.42	23.10 ± 2.67	24.12 ± 3.91	*t* = −1.25	0.217	23.44 (21.53, 24.92)	23.05 (20.70, 25.96)	Z = -0.07	0.944
Gender, *n* (%)				*χ*^2^ = 3.54	0.060			χ^2^ = 0.52	0.473
Female	33 (47.14)	19 (59.38)	14 (36.84)			18 (51.43)	15 (42.86)		
Male	37 (52.86)	13 (40.62)	24 (63.16)			17 (48.57)	20 (57.14)		
Smoking history, *n* (%)				*χ*^2^ = 5.22	0.022			*χ*^2^ = 0.00	1.000
0	40 (57.14)	23 (71.88)	17 (44.74)			20 (57.14)	20 (57.14)		
1	30 (42.86)	9 (28.12)	21 (55.26)			15 (42.86)	15 (42.86)		
Hypertension, *n* (%)				*χ*^2^ = 3.68	0.055			*χ*^2^ = 0.06	0.811
0	35 (50.00)	20 (62.50)	15 (39.47)			18 (51.43)	17 (48.57)		
1	35 (50.00)	12 (37.50)	23 (60.53)			17 (48.57)	18 (51.43)		
Diabetes, *n* (%)				*χ*^2^ = 2.79	0.095			*χ*^2^ = 0.08	0.771
0	55 (78.57)	28 (87.50)	27 (71.05)			27 (77.14)	28 (80.00)		
1	15 (21.43)	4 (12.50)	11 (28.95)			8 (22.86)	7 (20.00)		
Family history, *n* (%)				*χ*^2^ = 0.19	0.660			*χ*^2^ = 2.04	0.153
0	61 (87.14)	29 (90.62)	32 (84.21)			33 (94.29)	28 (80.00)		
1	9 (12.86)	3 (9.38)	6 (15.79)			2 (5.71)	7 (20.00)		
T stage, *n* (%)				*χ*^2^ = 0.13	0.719			*χ*^2^ = 0.00	1.000
T1	56 (80.00)	25 (78.12)	31 (81.58)			28 (80.00)	28 (80.00)		
T2/T3/T4	14 (20.00)	7 (21.88)	7 (18.42)			7 (20.00)	7 (20.00)		
N stage, *n* (%)				*χ*^2^ = 0.06	0.810			*χ*^2^ = 0.00	1.000
N0	63 (90.00)	28 (87.50)	35 (92.11)			31 (88.57)	32 (91.43)		
N1/N2/N3	7 (10.00)	4 (12.50)	3 (7.89)			4 (11.43)	3 (8.57)		
Patho, *n* (%)				*χ*^2^ = 1.44	0.231			*χ*^2^ = 0.09	0.759
Squamous cell lung cancer	13 (18.57)	4 (12.50)	9 (23.68)			7 (20.00)	6 (17.14)		
Adenocarcinoma	57 (81.43)	28 (87.50)	29 (76.32)			28 (80.00)	29 (82.86)		
CEA (ng/ml), M (Q₁, Q₃)	2.63 (1.78, 3.94)	2.14 (1.62, 3.10)	3.08 (1.92, 5.61)	*Z* = −2.17	0.030	2.66 (1.78, 3.40)	2.39 (1.81, 4.75)	*Z* = −0.19	0.849
SF (μg/L), M (Q₁, Q₃)	119.00 (69.75, 196.25)	130.00 (73.00, 185.00)	119.00 (70.00, 203.00)	*Z* = −0.25	0.801	109.00 (59.50, 180.00)	173.00 (100.00, 232.00)	*Z* = −1.80	0.071
NSE (ng/ml), M (Q₁, Q₃)	13.45 (10.67, 17.83)	13.90 (11.30, 17.85)	12.60 (10.30, 16.60)	*Z* = −0.71	0.475	14.40 (11.30, 18.60)	13.40 (10.60, 16.30)	*Z* = −0.82	0.411
CYFRA21-1 (ng/ml), M (Q₁, Q₃)	2.00 (1.36, 2.70)	1.59 (1.25, 2.50)	2.43 (1.45, 2.80)	*Z* = −1.91	0.056	2.47 (1.44, 3.14)	1.80 (1.32, 2.63)	*Z* = −1.72	0.085
SCCA (ng/ml), M (Q₁, Q₃)	0.47 (0.37, 0.65)	0.48 (0.38, 0.68)	0.44 (0.37, 0.59)	*Z* = −0.29	0.772	0.43 (0.35, 0.60)	0.49 (0.42, 0.64)	*Z* = −1.59	0.112

*t*, *t*-test; Z, Mann–Whitney test; *χ*^2^, Chi-square test; −, Fisher exact; SD, standard deviation; M, Median; Q₁, 1st Quartile; Q₃, 3st Quartile; CEA, carcinoembryonic antigen; SF, serum ferritin; NSE, neuron specific enolase; CYFRA21-1, cytokeratin fragment; SCCA, squamous cell carcinoma antigen.

### 3.2 SMA model construction and comparison

**Figure 4:**
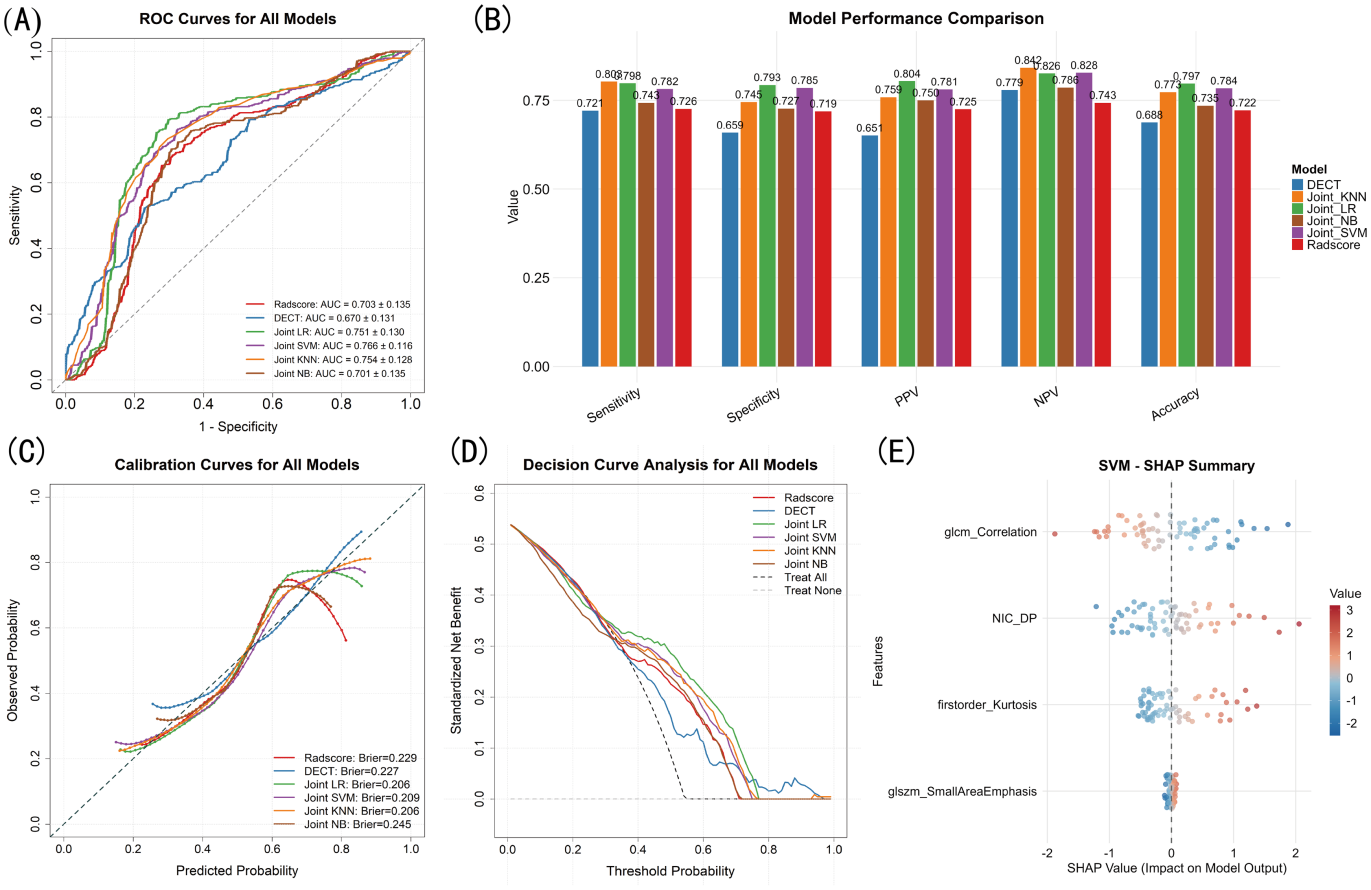
SMA model construction and comparison. **(A)** ROC for all models. **(B)** Model performance bar plots. **(C)** Calibration curves. **(D)** Decision curve analysis. **(E)** SHAP summary of SVM model.

Measurements of all DECT quantitative parameters were consistent between the two readers, as indicated by ICC values > 0.75 for each parameter (Supplementary S1.4). The characteristics of DECT parameters between groups with different levels of *α*-SMA expression are summarized in Table 2. The results showed that NIC(DP) and ECV were significantly higher in the SMA-high group than in the SMA-low group (all *p* < 0.05). Given the high correlation between NIC(DP) and ECV (*ρ* = 0.963, *p* < 0.001, Figure 2C), the NIC(DP) was ultimately kept after feature selection (Figure 2D). The AUC of NIC(DP) was 0.685 when used directly (Figure 2E) and 0.670 ± 0.131 after 5-fold CV in the LRA (Figure 4A).

**Table 2:** DECT parameters comparison in SMA-high and -low groups.

Variable	SMA-high	SMA-low	*P*
NIC(DP)	0.678 ± 0.249	0.528 ± 0.174	0.0054
ECV	0.351 (0.286, 0.444)	0.302 (0.238, 0.375)	0.0343
NIC(VP)	0.525 (0.416, 0.663)	0.462 (0.348, 0.547)	0.1967
Rho(DP)	−46.000 (−146.300, −17.875)	−84.700 (−323, −30.3)	0.1733
Zeff(DP)	8.325 (7.995, 8.633)	8.175 (7.042, 8.533)	0.1926
K_100-130_(DP)	−0.470 ± 0.181	−0.412 ± 0.161	0.1654
K_70-100_(DP)	−1.348 ± 0.512	−1.178 ± 0.459	0.1509
Rho(VP)	−54.350 (−202.450, −19.250)	−100.250 (−334.625, −33.075)	0.2291
Zeff(VP)	8.280 (7.965, 8.627)	8.255 (7.500, 8.535)	0.3162
IC(VP) (mg/ml)	2.050 (1.400, 2.475)	1.900 (1.300, 2.200)	0.3450
IC(DP) (mg/ml)	2.000 (1.500, 2.450)	1.700 (1.300, 2.250)	0.2989
K_100-130_(VP)	−0.475 (−0.588, −0.334)	−0.397 (−0.524, −0.320)	0.3734
K_40-70_(DP)	−1.355 (−1.650, −0.953)	−1.135 (−1.505, −0.919)	0.4092
K_40-70_(VP)	−4.628 (−6.024, −3.411)	−4.012 (−5.300, −3.411)	0.5957
K_70-100_(VP)	−4.172 (−6.019, −3.509)	−4.392 (−5.424, −3.236)	0.6039

For the *t*-test, the statistic is the mean ± standard deviation; for the Wilcoxon test, the statistic is the median (interquartile range); VP, venous phase; DP: delayed phase; K40–70 = (CT_70  keV_–CT_40 keV_)/30; K70–100 = (CT_100 keV_–CT_70keV_)/30; K100–130 = (CT_130 keV_–CT_100keV_)/30; Rho: electron cloud density; Zeff, effective atomic number; (N)IC, (normalized) iodine concentration; ECV(%) = (1-hematocrit) × NIC.

Following the same feature selection process, three radiomics features (glcm_Correlation, firstorder_Kurtosis and glszm_SmallAreaEmphasis) were selected, and the AUC was 0.703 ± 0.135 after 5-fold CV of LRA. Radiomics model demonstrated higher SEN, SPE, PPV, NPV and ACC (Figure 4B). Although there was no significant difference in AUC compared to DECT model (Table 3), the calibration curve indicated that DECT model had better agreement between predicted probabilities and observed outcomes (Figure 4C).

**Table 3:** SMA models comparison.

Comparison	*t*_stat	df	*P*	*P*.adj
Radscores vs. DECT	1.4989	49	0.1403	1
Radscores vs. Joint_LR	−5.0234	49	0	1e-04
Radscores vs. Joint_SVM	−5.7026	49	0	0
Radscores vs. Joint_KNN	−5.0456	49	0	1e-04
Radscores vs. Joint_NB	0.1994	49	0.8427	1
DECT vs. Joint_LR	−3.9775	49	2e-04	0.0034
DECT vs. Joint_SVM	−4.6962	49	0	3e-04
DECT vs. Joint_KNN	−3.5833	49	8e-04	0.0117
DECT vs. Joint_NB	−1.3486	49	0.1837	1

DECT, dual energy computed tomography; LR, logistic regression; SVM, support vector machine; KNN, K-nearest neighbors; NB, naive bayes; t_stat, t statistics; df, degree of freedom; *P*.adj, false discovery rate.

Then we integrated all selected features to construct models, where joint LR (AUC = 0.751 ± 0.130), joint SVM (AUC = 0.766 ± 0.116) and joint KNN (AUC = 0.754 ± 0.128) showed significant improvements than single mode models (Table 3) with smaller brier scores, wider threshold as well as higher net benefit (Figures 4C,D). We illustrated the feature contribution of joint SVM model using SHAP summary plot (Figure 4E), and only the glcm_Correlation had negative effects.

### 3.3 CD8 + T cells model construction and comparison

**Figure 5:**
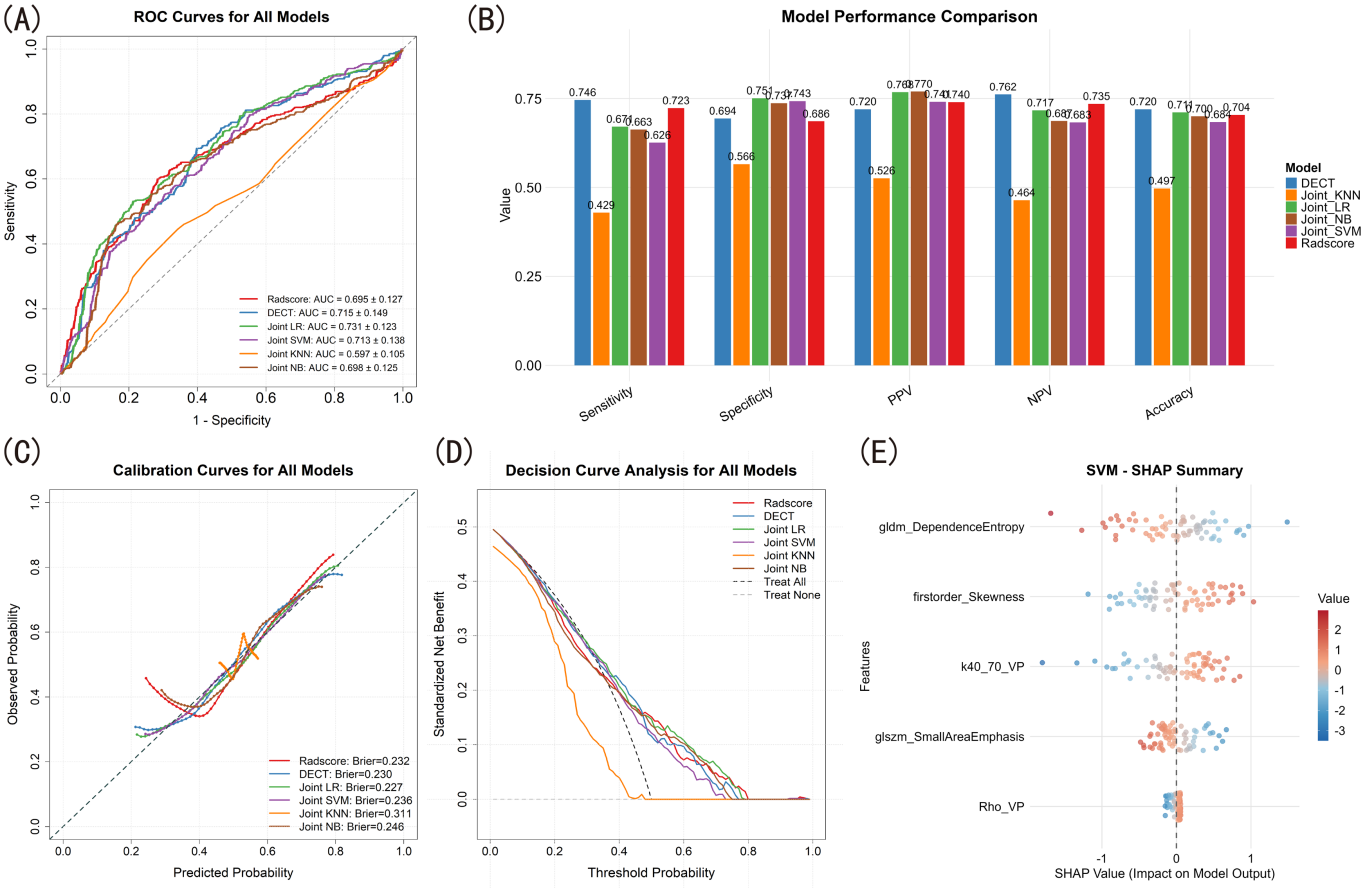
CD8+T cells model construction and comparison. **(A)** ROC for all models. **(B)** Model performance bar plots. **(C)** Calibration curves. **(D)** Decision curve analysis. **(E)** SHAP summary of SVM model.

The characteristics of DECT parameters between groups with different levels of CD8 expression showed that most parameters were significantly different between CD8-high and CD8-low group (Table 4). After feature selection, K40-70(VP) and Rho(VP) were conserved (Figure 3C,D), yielding AUCs of 0.701 and 0.640, respectively, (Figure 3E). Patients in CD8-high group had higher K40-70(VP) and lower Rho(VP) values. When simply combined by LRA, the DECT model got an AUC of 0.715 ± 0.149 (Figure 5A).

**Table 4:** DECT parameters comparison in CD8-high and -low groups.

Variable	CD8-high	CD8-low	*P*
Z(VP)	7.980 (7.025, 8.325)	8.570 (8.160, 8.860)	0.0002
Z(DP)	8.110 (6.735, 8.325)	8.490 (8.080, 8.780)	0.0011
K_40-70_(VP)	−3.677 (−5.103, −3.112)	−5.213 (−6.242, −3.912)	0.0039
IC(VP) (mg/ml)	1.700 (1.200, 2.050)	2.100 (1.750, 2.650)	0.0089
K_100-130_(VP)	−0.377 (−0.525, −0.297)	−0.513 (−0.628, −0.377)	0.0075
K_40-70_(DP)	−1.083 (−1.500, −0.843)	−1.470 (−1.790, 1.072)	0.0087
ECV	0.291 (0.236, 0.357)	0.359 (0.284, 0.439)	0.0485
NIC(DP)	0.512 (0.421, 0.637)	0.636 (0.494, 0.745)	0.0458
NIC(VP)	0.426 (0.302, 0.579)	0.511 (0.439, 0.674)	0.0433
Rho(VP)	−99.000 (−424.500, −41.050)	−41.900 (−111.140–23.250)	0.0440
Rho(DP)	−128.300 (−411.700, −42.100)	−45.700 (−102.400, −18.000)	0.0398
K_70-100_(VP)	−4.020 (−4.675, −3.060)	−5.133 (−6.282, −3.618)	0.0298
K_100-130_(DP)	−0.407 (−0.485, −0.335)	−0.473 (−0.568, −0.350)	0.1297
K_70-100_(DP)	−1.157 (−1.387, −0.957)	−1.350 (−1.628, −1.008)	0.1239
IC(DP) (mg/ml)	1.800 (1.250, 2.350)	2.000 (1.550, 2.350)	0.1724

For the *t*-test, the statistic is the mean ± standard deviation; for the Wilcoxon test, the statistic is the median (interquartile range); VP, venous phase; DP: delayed phase; K40–70 = (CT_70  keV_–CT_40 keV_)/30; K70–100 = (CT_100 keV_–CT_70keV_)/30; K100–130 = (CT_130 keV_–CT_100keV_)/30; Rho: electron cloud density; Zeff, effective atomic number; (N)IC, (normalized) iodine concentration; ECV(%) = (1-hematocrit) × NIC.

Three radiomic features (gldm_DependenceEntropy, firstorder_Skewness and glszm_Small-AreaEmphasis) were conserved in CD8 + T cells prediction, and the AUC of radiomics model was 0.695 ± 0.127 after 5-fold CV of LRA. DECT model showed better performance under this condition (Figure 5B), although again no significant difference was observed in AUC compared to radiomics model (Table 5). The calibration curve indicated that DECT model maintained better agreement between predicted probabilities and observed outcomes (Figure 5C). DCA revealed that all models except KNN demonstrated clinical utility (Figure 5D).

**Table 5:** CD8 models comparison.

Comparison	t_stat	df	*P*	*P*.adj
Radscores vs. DECT	−0.8579	49	0.3951	1
Radscores vs. Joint_LR	−1.8107	49	0.0763	1
Radscores vs. Joint_SVM	−1.7664	49	0.0836	1
Radscores vs. Joint_KNN	4.9219	49	0	2e-04
Radscores vs. Joint_NB	2.5911	49	0.0126	0.1886
DECT vs. Joint_LR	−0.2819	49	0.7792	1
DECT vs. Joint_SVM	−0.2242	49	0.8235	1
DECT vs. Joint_KNN	5.3731	49	0	0
DECT vs. Joint_NB	3.1987	49	0.0024	0.0363

DECT, dual energy computed tomography; LR, logistic regression; SVM, support vector machine; KNN, K-nearest neighbors; NB, naive bayes; t_stat, t statistics; df, degree of freedom; *P*.adj, false discovery rate.

However, the joint models showed no significant improvement with single mode models (Table 5). The feature contribution was also shown by SHAP summary plot (Figure 5E).

## 4 Discussion

This study aimed to non-invasively characterize key components of the TME in NSCLC by integrating DECT parameters and radiomics features. We successfully developed and validated models for predicting the expression levels of *α*-SMA and tumor-infiltrating lymphocytes (CD8 + T cells). The integrated model incorporating both DECT and radiomics demonstrated improved predictive performance for differentiating SMA-high from SMA-low tumors. In contrast, for CD8 + T-cell stratification, the DECT-only model yielded comparable discriminative ability. These findings highlight the potential of a non-invasive, imaging-based approach to decode the complex cellular composition of the NSCLC TME.

The predictive value of DECT parameters stems from their ability to reflect underlying tissue composition and physiology. For instance, IC showed a significant positive correlation with density of microvascular marked by CD34 in ccRCC (21), while ECV has been shown to be significantly elevated in fibrotic diseases and various tumors, suggesting ongoing ECM remodeling (22–24). In our study, the significantly higher NIC(DP) and ECV in the SMA-high group align with this established association between ECV and stromal fibrosis, thereby reinforcing their potential as imaging surrogates for CAF-driven ECM remodeling. For predicting CD8 + T-cell density, K40-70(VP) and Rho(VP) were ultimately selected. Previous research has utilized the absolute slope of energy spectral attenuation curve and Rho to differentiate tissue composition between benign and malignant lesions, reporting higher values in more aggressive tumors (25, 26). While this suggests a link to tissue heterogeneity and cellularity, the precise biological mechanisms under-pinning the relationship between these specific spectral parameters and immune cell infiltration require further elucidation. Historically, DECT applications in NSCLC have predominantly focused on lesion characterization, histological subtyping, and prognostic assessment (27–29). More recently, the scope has expanded to include the prediction of key molecular markers such as programmed death ligand 1 (PD-L1), Ki-67, and thyroid transcription factor 1 (TTF-1) (30, 31). Our work builds upon and extends this evolving paradigm by shifting the focus to direct, cellular-level constituents of the TME, specifically CAFs and cytotoxic T lymphocyte, thus confirming and broadening the potential of DECT-derived metrics to decode the tumor immune landscape.

The capability of radiomics to characterize TME has been demonstrated by an expanding body of research (32). In our study, the radiomics models for predicting the expression of *α*-SMA and CD8 + T cells yielded AUCs of 0.703 and 0.695, respectively. Notably, while their performance was not statistically inferior to that of the DECT-based models, the feature glszm_SmallAreaEmphasis was consistently selected in both models. SHAP analysis revealed that this feature exerted opposing directional effects in the two models, a finding that aligns with the antagonistic biological roles of α-SMA + CAFs and CD8 + T cells within the TME. This oppsition may reflect distinct spatial distributions, in α-SMA-high tumors, a higher value may reflect a multifocal or compartmentalized process of fibrosis, where the stroma is not a single, confluent mass but consists of multiple smaller, dense fibrotic regions interspersed within cellular-rich areas (33). Conversely, in CD8-high tumors, a lower value may suggest more extensive intratumoral structural disruption (e.g., necrosis) or greater overall heterogeneity, where these macroscopic alterations obscure the fine-grained textural signal contributed by the lymphocyte clusters themselves. Consistently, the SMA-high group exhibited imaging phenotypes suggestive of greater textural heterogeneity (lower glcm_Correlation, higher firstorder_Kurtosis). Conversely, the CD8-high group was associated with higher skewness (hinting at lymphoid clusters) and lower gldm_DependenceEntropy, implying tissue homogeneity from dense CD8 + T-cell infiltration.

The integrated model combining selected DECT parameters and radiomics features developed in this study achieved the best performance for predicting *α*-SMA expression, significantly outperforming models using either modality alone. This synergy suggests that macroscopic functional information from DECT and microscopic textural heterogeneity from radiomics are complementary for characterizing stromal abundance. In contrast, for predicting CD8 + T-cell density, the combined model did not yield a statistically significant improvement over simpler models. This differential gain may be attributed to the distinct biological nature of the targets. ECM remodeling may manifest more distinctly in both tissue composition (captured by DECT) and architectural distortion (captured by radiomics), whereas lymphocytic infiltration might be more directly reflected in functional perfusion changes or require more specific feature classes for optimal radiomics capture such as wavelet transforming (32). While readily available, systemic inflammatory indices like NLR did not correlate with our pathological TME readouts in this cohort, suggesting that the DECT parameters and radiomics features capture distinct, tissue-localized biological information. Although the AUC values of our models (0.73–0.76) are modest and may appear lower than some studies predicting single protein biomarkers like PD-L1 using DECT (e.g., AUC = 0.83) (31), our study pioneers the non-invasive and comprehensive assessment of cellular constituents within the TME, which represents a more complex biological task.

This study has several limitations. Firstly, due to the requirement for pathological confirmation, the study was conducted on a relatively small, single-center cohort. Future work should include validation in larger, multi-center populations. In addition, the clinical utility of this integrated approach warrants further evaluation in prospective studies investigating response to immunotherapy or chemotherapy. Secondly, our analysis was restricted to 2D ROIs, which limits comprehensive assessment of volumetric heterogeneity. Future efforts should focus on 3D whole-tumor analysis of DECT parameter maps and seek to correlate these imaging features with spatially resolved molecular data (e.g., transcriptomics), thereby grounding radiomic features in explicit biology. Lastly, the timing of the delayed phase for ECV calculation was not standardized, which may affect reproducibility and requires further validation.

## 5 Conclusion

In conclusion, by integrating DECT parameters and radiomics features, this study provides a novel, non-invasive framework for evaluating the complexity of the NSCLC TME. It highlights that while these modalities are complementary, their relative importance varies across TME components, offering a refined path toward developing actionable imaging biomarkers for personalized oncology.

## Data Availability

The raw data supporting the conclusions of this article will be made available by the authors, without undue reservation.

## Footnotes

1
https://www.slicer.org/
