# Supplementary material for: Integration of dual-energy CT parameters and radiomics features for non-invasive prediction of α-SMA and CD8 + T cell in non-small cell lung cancer
Source: Front Med (Lausanne). 2026 Mar 11;13:1792692. doi: 10.3389/fmed.2026.1792692 (PMC13013019; doi:10.3389/fmed.2026.1792692)
Supplement: Supplementary file 1 [file Table_1.DOCX]

**Supplementary Materials**

1. **Dual-Energy CT scanning protocols and acquisition parameters**

All patients underwent thoracic scanning with a dual-source CT scanner (SOMATOM Force; Siemens Healthcare, Forchheim, Germany). Dual-energy acquisition was performed only during the venous phase (VP, t = 65 s) and the delayed phase (DP, t = 120 s). Patients were positioned supine and scanned at the end of deep inspiration, covering the lung volume from the apex to the base. For the examination, a non-ionic iodinated contrast agent (iomeprol injection, 400 mgI/mL; Bracco, Italy) was administered intravenously as a bolus at a dose of 1.0-1.5 mL/kg (not exceeding 65 mL), with an injection flow rate of 3.0 mL/s, followed by a 30-mL saline flush at the same flow rate. The tube voltages for tubes A and B were set at 90 kV and Sn150 kV, respectively. The quality reference mAs was 150/115 mAs, the rotation time was 0.25 s, the pitch was 1.2 and images were reconstructed with a slice thickness of 1 mm, a slice increment of 1 mm, and a convolution kernel of Qr69.

There was no standard delayed phase time for chest CT. Prior dynamic contrast-enhanced CT studies in lung diseases use delays ranging from 90 seconds for differentiating lung nodules[1], to even 5 minutes for assessing late enhancement in idiopathic pulmonary fibrosis (IPF)[2]. Our choice of 120 seconds is supported by several factors: (1) Biological & Technical Basis: 5-min delay expiration scan was used for late enhancement imaging as a surrogate marker for slow washout of contrast media of the lung parenchyma due to alveolar-capillary leakage and increased interstitial volume in IPF. However, NSCLC tumors typically do not reach the same degree of diffuse fibrosis as seen in IPF. Therefore, an excessively long delay may not be necessary and could increase the risk of patient motion or contrast washout heterogeneity. (2) Practical Clinical Integration: A 120-second delay fits efficiently within a routine clinical dual-phase chest CT protocol (e.g., venous phase at 60-70s, delayed phase at 120s), enhancing the translational potential of our approach without unduly prolonging scan time or contrast dose. (3) Support from Pre-experimental Data: Prior to finalizing our protocol, we conducted a preliminary analysis comparing ECV values derived from scans at 90 seconds and 120 seconds post-injection in a subset of our cohort. We observed that ECV values measured at 120 seconds were consistently and significantly higher than those at 90 seconds, suggesting that the later time point better captured the contrast equilibrium in the tumor interstitium, likely providing a more accurate estimate of the true extracellular space.

1. **Pathological analysis**

Quantitative analysis of immunohistochemically stained sections was performed using ImageJ software (Version 1.54). Custom macro scripts were employed to enable batch processing and reproducible image handling and data extraction. The analysis focused on two markers: α-smooth muscle actin (α-SMA) and CD8. Both were developed with DAB (brown) chromogen and hematoxylin (blue) counterstain; the color channel corresponding to DAB was targeted for analysis. The final results were verified by an experienced pathologist.

1. **Analysis of α-SMA-Positive Area Percentage**

α-SMA exhibits continuous or sheet-like expression within the tumor stroma; therefore, it was quantified using the percentage of positive area. For α-SMA, the positive area was quantified specifically within the tumor stromal compartment. Areas of tumor cells displaying positive cytoplasmic staining (e.g., in sarcomatoid components) were excluded from the analysis to focus specifically on cancer-associated fibroblast activity. The analytical procedure was as follows: First, the RGB image was split into red, green, and blue color channels, and the blue channel complementary to the DAB signal was selected for subsequent processing. To accurately calculate the actual tissue area, a high grayscale threshold (235–255) was set to identify and exclude the white background of the image. Subsequently, a specific grayscale threshold (20–140) was applied to distinguish positively stained regions from negative tissue. The software calculated the total tissue area (Tissue Area) after background subtraction and the area within the threshold (Positive Area). The final output was the percentage of the positive area relative to the total tissue area (Percentage). The analysis simultaneously generated an overlay image for visual verification, where positive areas were marked in red and the background was marked in black.


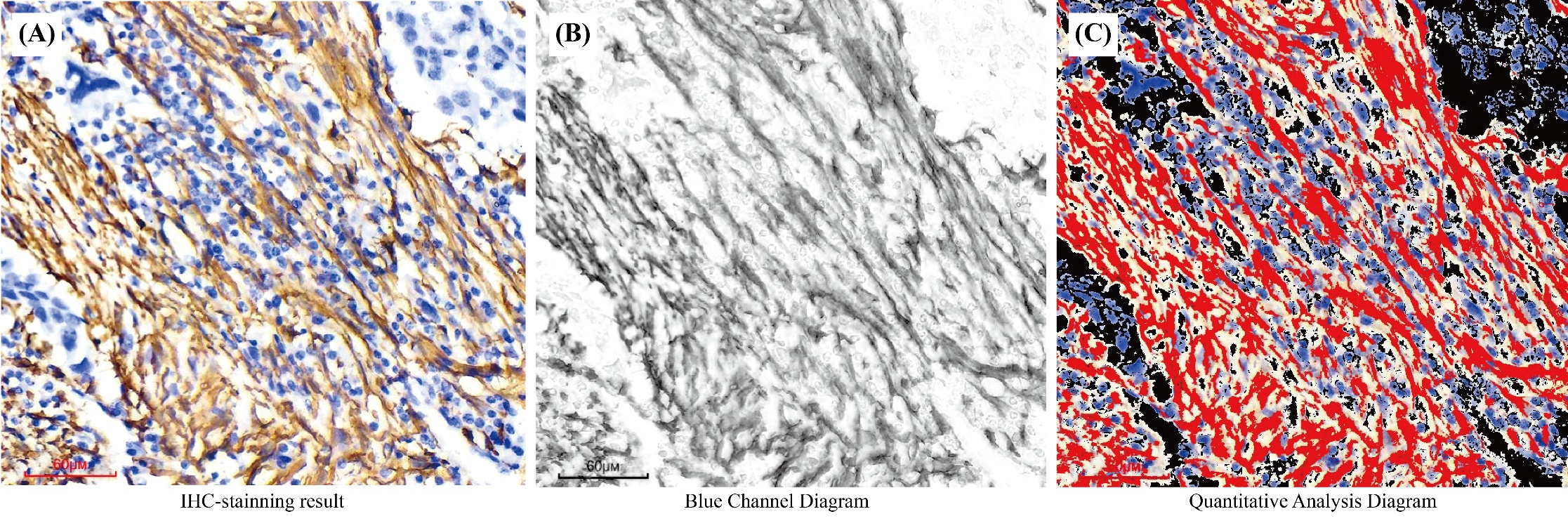


Figure S1. Analysis of α-SMA-Positive Area Percentage. (A) Original IHC-staining result. (B) Blue-channel diagram for showing positive α-SMA. (C) Overlay image for visual verification.

1. **Analysis of CD8+T Lymphocyte Count and Density**

CD8+ T lymphocytes are discretely distributed round cells; therefore, they were quantified by cell counting and density. The analysis consisted of two main steps: tissue area identification and positive cell identification. First, the original image was converted to an 8-bit grayscale image. The entire tissue region was defined by setting a grayscale threshold (0–235) and applying the "Analyze Particles" function to calculate its area (Tissue Area). Subsequently, for the split blue channel image, a threshold (20–140) was set to initially select positive signals, and the "Convert to Mask" function was used to create a binary image. To separate closely adjacent cells, the "Watershed" algorithm was applied to the binary image for segmentation. Finally, the "Analyze Particles" function (with the particle size range set from 100 pixels to infinity [3]) was used to identify and count the segmented individual particles, outputting the total number of positive cells (Positive Cell Count). The cell density (Positive Cell Density) was calculated by dividing the total positive cell count by the tissue area. The analysis process generated a composite image displaying the tissue area (overlaid in blue) and positive cells (overlaid in red) for result verification. The final counts were reviewed and manually adjusted by the pathologist.


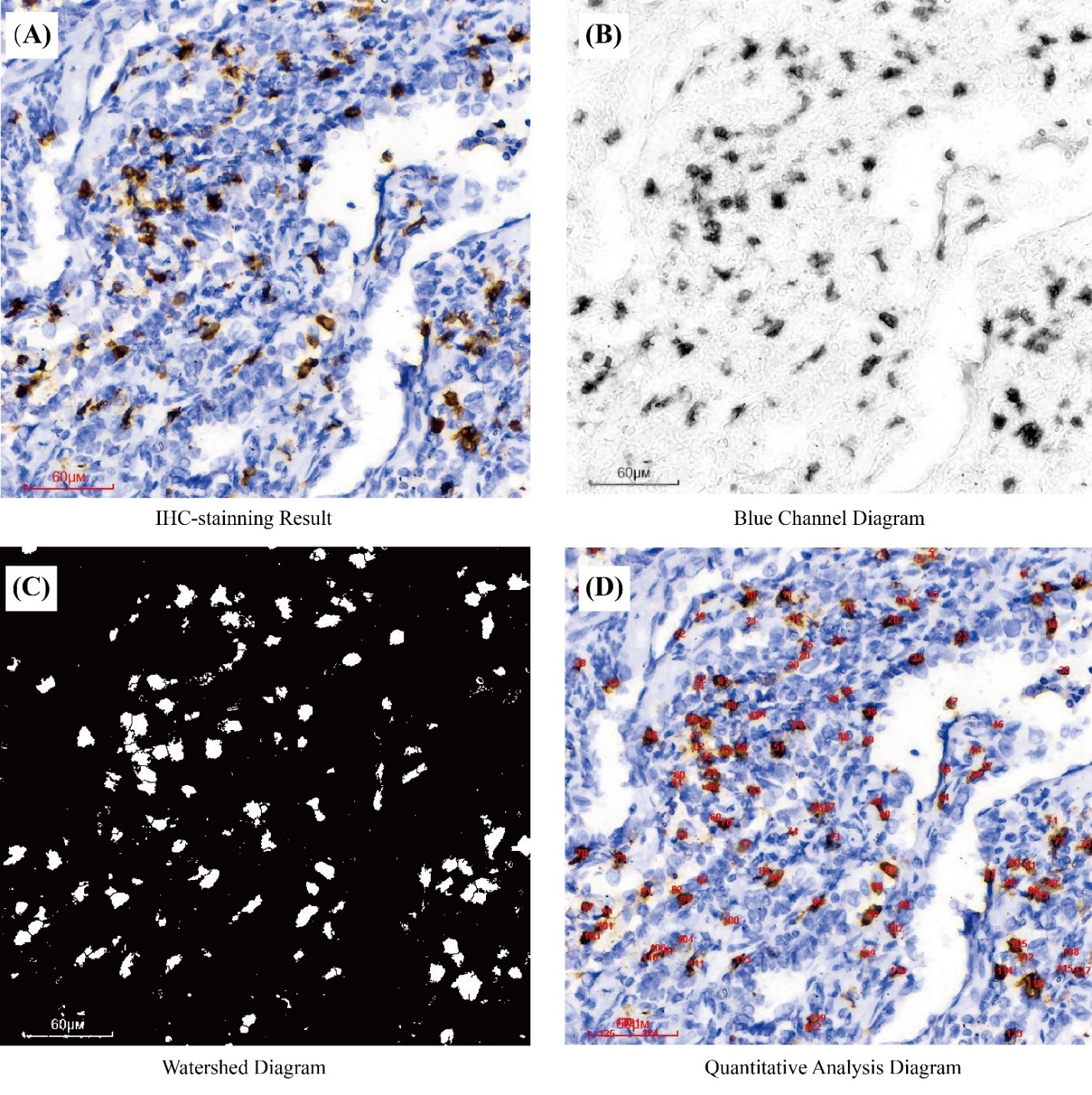


Figure S2. Analysis of CD8+T Lymphocyte Count. (A) Original IHC-staining result. (B) Blue-channel diagram for showing positive cells. (C) Watershed diagram for counting cells. (D) Quantitative analysis diagram for verification.

1. **The distribution of SMA and CD8**

The distribution of average percentage of α-SMA-Positive Area and CD8 are shown in the figure. In the analysis, we use different thresholds for binary classification since the SMA area proportion was normally distributed and the CD8+ T-cell density was skewed distribution, which is as same as previous studies [3].


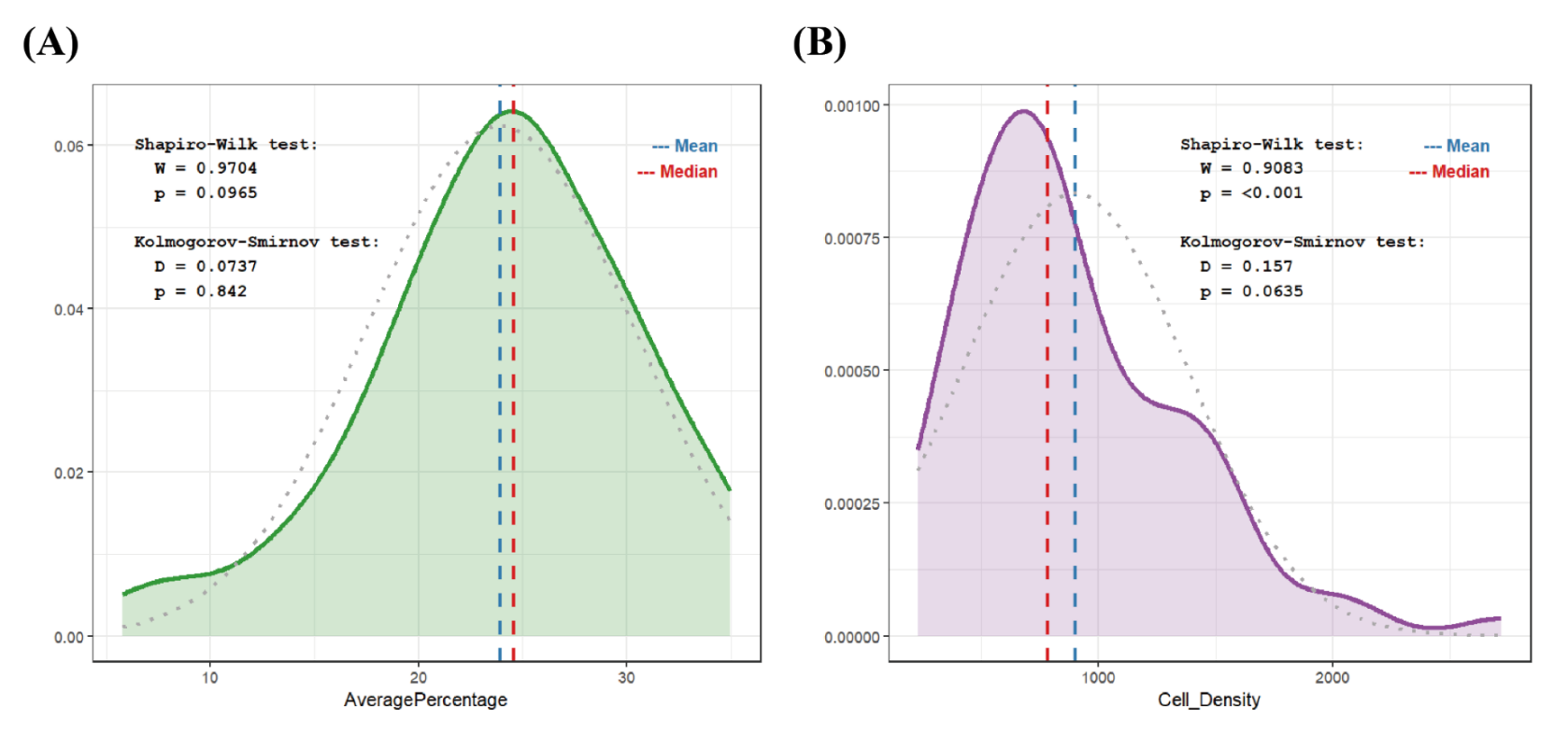


Figure S3. Distribution and normality test. (A) α-SMA-Positive Area percentage. (B) CD8+T Cell density.

1. **ICC results of DECT parameters**

A total of 15 features were measured from each tumor’s region of interest. ICC results are as follows.

| **Feature** | **ICC** | **Feature** | **ICC** |
| --- | --- | --- | --- |
| IC(VP) | 0.8515 | IC(DP) | 0.9048 |
| NIC(VP) | 0.8341 | NIC(DP) | 0.9548 |
| Rho(VP) | 0.9959 | Rho(DP) | 0.9869 |
| Zeff(VP) | 0.8797 | Zeff(DP) | 0.9033 |
| K_40-70_(VP) | 0.9860 | K_40-70_(DP) | 0.7934 |
| K_70-100_(VP) | 0.9801 | K_70-100_(DP) | 0.8145 |
| K_100-130_(VP) | 0.9388 | K_100-130_(DP) | 0.8099 |
| ECV | 0.9548 |  |  |

ICC: intra-class correlation coefficients; VP: venous phase; DP: delayed phase; ECV: extracellular volume.

1. **ICC results of radiomic features**

A total of 93 features were extracted from each tumor’s region of interest. ICC results are as follows.

| **Feature** | **ICC** | **Feature** | **ICC** |
| --- | --- | --- | --- |
| firstorder_10Percentile | 0.9919 | gldm_GrayLevelVariance | 0.9544 |
| firstorder_90Percentile | 0.9995 | gldm_HighGrayLevelEmphasis | 0.8937 |
| firstorder_Energy | 0.9663 | gldm_LargeDependenceEmphasis | 0.9882 |
| firstorder_Entropy | 0.9551 | gldm_LargeDependenceHighGrayLevelEmphasis | 0.9131 |
| firstorder_InterquartileRange | 0.9722 | gldm_LargeDependenceLowGrayLevelEmphasis | 0.7092 |
| firstorder_Kurtosis | 0.9645 | gldm_LowGrayLevelEmphasis | 0.5459 |
| firstorder_Maximum | 0.9847 | gldm_SmallDependenceEmphasis | 0.9741 |
| firstorder_MeanAbsoluteDeviation | 0.9600 | gldm_SmallDependenceHighGrayLevelEmphasis | 0.9253 |
| firstorder_Mean | 0.9979 | gldm_SmallDependenceLowGrayLevelEmphasis | 0.5867 |
| firstorder_Median | 0.9981 | glrlm_GrayLevelNonUniformity | 0.9995 |
| firstorder_Minimum | 0.9500 | glrlm_GrayLevelNonUniformityNormalized | 0.9627 |
| firstorder_Range | 0.8633 | glrlm_GrayLevelVariance | 0.9528 |
| firstorder_RobustMeanAbsoluteDeviation | 0.9664 | glrlm_HighGrayLevelRunEmphasis | 0.8926 |
| firstorder_RootMeanSquared | 0.9963 | glrlm_LongRunEmphasis | 0.9867 |
| firstorder_Skewness | 0.9825 | glrlm_LongRunHighGrayLevelEmphasis | 0.8895 |
| firstorder_TotalEnergy | 0.9685 | glrlm_LongRunLowGrayLevelEmphasis | 0.5542 |
| firstorder_Uniformity | 0.9664 | glrlm_LowGrayLevelRunEmphasis | 0.5406 |
| firstorder_Variance | 0.9540 | glrlm_RunEntropy | 0.9391 |
| glcm_Autocorrelation | 0.8963 | glrlm_RunLengthNonUniformity | 0.9990 |
| glcm_ClusterProminence | 0.9712 | glrlm_RunLengthNonUniformityNormalized | 0.9856 |
| glcm_ClusterShade | 0.9764 | glrlm_RunPercentage | 0.9864 |
| glcm_ClusterTendency | 0.9709 | glrlm_RunVariance | 0.9872 |
| glcm_Contrast | 0.9715 | glrlm_ShortRunEmphasis | 0.9854 |
| glcm_Correlation | 0.9745 | glrlm_ShortRunHighGrayLevelEmphasis | 0.8942 |
| glcm_DifferenceAverage | 0.9758 | glrlm_ShortRunLowGrayLevelEmphasis | 0.5381 |
| glcm_DifferenceEntropy | 0.9684 | glszm_GrayLevelNonUniformity | 0.9995 |
| glcm_DifferenceVariance | 0.9636 | glszm_GrayLevelNonUniformityNormalized | 0.8890 |
| glcm_Id | 0.9815 | glszm_GrayLevelVariance | 0.9231 |
| glcm_Idm | 0.9822 | glszm_HighGrayLevelZoneEmphasis | 0.8790 |
| glcm_Idmn | 0.9308 | glszm_LargeAreaEmphasis | 0.9987 |
| glcm_Idn | 0.9120 | glszm_LargeAreaHighGrayLevelEmphasis | 0.9534 |
| glcm_Imc1 | 0.9887 | glszm_LargeAreaLowGrayLevelEmphasis | 0.9615 |
| glcm_Imc2 | 0.9913 | glszm_LowGrayLevelZoneEmphasis | 0.4673 |
| glcm_InverseVariance | 0.9752 | glszm_SizeZoneNonUniformity | 0.9983 |
| glcm_JointAverage | 0.8804 | glszm_SizeZoneNonUniformityNormalized | 0.9011 |
| glcm_JointEnergy | 0.9780 | glszm_SmallAreaEmphasis | 0.8830 |
| glcm_JointEntropy | 0.9704 | glszm_SmallAreaHighGrayLevelEmphasis | 0.8868 |
| glcm_MCC | 0.9397 | glszm_SmallAreaLowGrayLevelEmphasis | 0.4822 |
| glcm_MaximumProbability | 0.9793 | glszm_ZoneEntropy | 0.9367 |
| glcm_SumAverage | 0.8804 | glszm_ZonePercentage | 0.9817 |
| glcm_SumEntropy | 0.9709 | glszm_ZoneVariance | 0.9987 |
| glcm_SumSquares | 0.9704 | ngtdm_Busyness | 0.9643 |
| gldm_DependenceEntropy | 0.9378 | ngtdm_Coarseness | 0.9844 |
| gldm_DependenceNonUniformity | 0.9991 | ngtdm_Complexity | 0.9260 |
| gldm_DependenceNonUniformityNormalized | 0.9894 | ngtdm_Contrast | 0.9540 |
| gldm_DependenceVariance | 0.9914 | ngtdm_Strength | 0.9673 |
| gldm_GrayLevelNonUniformity | 0.9995 |  |  |

ICC: intra-class correlation coefficients.

1. **The analyses of systemic inflammatory indices**

The analysis demonstrated no statistically significant correlations (all P > 0.05, Spearman's rank test) between the systemic inflammatory indices (NLR, PLR) and the pathological biomarkers (α-SMA, CD8). Furthermore, these indices showed limited discriminative capacity for predicting TME status in our cohort, with all AUC values below 0.6.

Table S1 The correlation between systemic inflammatory indices and the target pathological biomarkers

| Pathological biomarker | Parameter | ρ | *P* | AUC (95% CI) |
| --- | --- | --- | --- | --- |
| α-SMA | NLR | 0.007 | 0.956 | 0.519 (0.372, 0.666) |
|  | PLR | 0.084 | 0.499 | 0.531 (0.393, 0.669) |
| CD8 | NLR | 0.007 | 0.956 | 0.519 (0.372, 0.666) |
|  | PLR | 0.084 | 0.499 | 0.531 (0.393, 0.669) |

NLR: neutrophil-to-lymphocyte ratio, PLR: latelet-to-lymphocyte ratio, AUC: area under the curve.

1. Jiang X A, Ma Q, Zhou T, Feng Q, Yang W, Zhou X, et al. Extracellular volume fraction as a potential predictor to differentiate lung cancer from benign lung lesions with dual-layer detector spectral CT. Quantitative Imaging In Medicine and Surgery (2023) 13(12): 8121–8131. doi: 10.21037/qims-23-736

2. Scharm S C, Vogel-Claussen J, Schaefer-Prokop C, Dettmer S, Knudsen L, Jonigk D, et al. Quantification of dual-energy CT-derived functional parameters as potential imaging markers for progression of idiopathic pulmonary fibrosis. European Radiology (2021) 31(9): 6640–6651. doi: 10.1007/s00330-021-07798-w

3. Lin H, Pan X, Feng Z, Yan L, Hua J, Liang Y, et al. Automated whole-slide images assessment of immune infiltration in resected non-small-cell lung cancer: towards better risk-stratification. Journal of Translational Medicine (2022) 20(1): 261. doi: 10.1186/s12967-022-03458-9
